# Hydrodynamic repulsion of spheroidal microparticles from micro-rough surfaces

**DOI:** 10.1371/journal.pone.0183093

**Published:** 2017-08-14

**Authors:** Aleksey V. Belyaev

**Affiliations:** 1 Faculty of Physics, M.V.Lomonosov Moscow State University, 119991 Moscow, Russia; 2 Dmitry Rogachev National Research Center of Pediatric Hematology, Oncology and Immunology, 1 Samora Machel str., 117997 Moscow, Russia; 3 Center for Theoretical Problems of Physico-Chemical Pharmacology RAS, 38A Leninsky prospect, 119991 Moscow, Russia; Virginia Commonwealth University, UNITED STATES

## Abstract

Isolation of microparticles and biological cells from mixtures and suspensions is a central problem in a variety of biomedical applications. This problem, for instance, is of an immense importance for microfluidic devices manipulating with whole blood samples. It is instructive to know how the mobility and dynamics of rigid microparticles is altered by the presence of micrometer-size roughness on walls. The presented theoretical study addresses this issue via computer simulations. The approach is based on a combination of the Lattice Boltzmann method for calculating hydrodynamics and the Lagrangian Particle dynamics method to describe the dynamics of cell membranes. The effect of the roughness on the mobility of spheroidal microparticles in a shear fluid flow was quantified. We conclude that mechanical and hydrodynamic interactions lift the particles from the surface and change their mobility. The effect is sensitive to the shape of particles.

## Introduction

Particle suspensions can be found in many industrial, geophysical, and biological applications. Understanding the dynamics of a single particle in a linear shear flow provides fundamental knowledge of suspension flows. Hydrodynamic forces and torques acting on small particles enclosed by fluids depend on many factors, e.g., the local undisturbed flow field, fluid and particle inertia, external forces and torques, and particle shape. Also, the complex experimentally observed dynamics of red blood cells in viscous shear flows [1] demonstrates the importance of blood cell deformability. The effects related to fluid to membrane elasticity and interior-to-exterior viscosity ratio have been addressed in a number of theoretical works [2–5]. This effect is interconnected with important biomechanical phenomena like the Fahraeus and Fahraeus-Lindqvist effects (caused by formation of RBC-depleted layer near wall).

### Non-sphericity

The anisotropic orientation of nonspherical particles becomes an important factor, in particular, the rotational behaviour due to local velocity gradients determines the rheology of the suspension [6], especially in the vicinity of solid walls. For instance, nonspherical particles can move along fairly complicated trajectories, which are significantly affected by their shape and orientation. Moreover, non-neutrally buoyant nonspherical particles may drift in a gravity field. Sometimes this drift can be accounted for as a drift velocity of a small inertia-less particle, and be added to the fluid velocity when modelling particle transport [7].

The first studies of the rotational behaviour of spheroids in a Stokes flow were done analytically by Jeffery [8]. The ellipsoidal particle was found to undergo a periodic revolution called the Jeffery orbit, which is controlled by the anisotropy (aspect ratio) of the particle. The analytical solution for the Jeffery orbit was derived by Jeffery himself and soon experimentally verified by Taylor [9]. Jeffery showed that the angular motion of a prolate spheroid follows certain closed, periodic orbits that depend on the shear rate and the aspect ratio of the spheroid. Jeffery’s analysis was later proven valid for any axisymmetric particle in shear flow [10]. Later investigations on the Jeffery orbit involved wall effects [11–15], inertial effect [7, 16] and Poiseuille flow [17, 18].

### Inertia

In the absence of fluid and particle inertia, a spheroid was found to rotate in one of an infinite number of orbits. Effects of particle inertia are manifested in orientation of the particle so that it starts spinning around its axis of symmetry [6]. During the transition the particle performs both precession and nutation around the vorticity direction [6]. Jeffery [8] hypothesized that the influence of fluid inertia will make the particle rotate in the orbit corresponding to the least dissipation of energy. For the oblate particle, this means that it should rotate around its major axis, which is parallel to the vorticity axis in a rotational state called tumbling, and this hypothesis was confirmed later [9, 17, 19]. Another work [7] reports the motion of prolate spheroids in shear flow near wall in cases, when the particle density is big compared to fluid. The margination of particles towards the wall was observed in numerical solutions attributed only to particle inertia, while fluid inertia was negligible.

### Near-wall inertial lift

Small particles can experience lift forces as a result of fluid inertia. Lift forces can cause particles to cross streamlines in laminar flows. There is a number of engineering applications where lift forces are known or suspected to play an important role.

Slow motion of neutrally buoyant particles in tubes and channels may be treated as occurring along the flow streamlines. This, however, is not true even for spheres, which migrate across the streamlines as a result of fluid inertia. Nowadays it is well known that, even at very small Reynolds number, a particle moving in a shear flow experiences a lift force, the origin of which is inertia effects see, for example, the well-known experimental results of Segre and Silberberg. By investigating the steady motion of a sphere in a shear flow Saffman [20] obtained the expression of the lift force acting on the sphere. It was assumed that the migration velocity in unbounded shear flow is small enough that it may be neglected. However, wall-induced effects are more than important in suspension flows, e.g. the formation of erythrocyte-free layer in microvessels, known as the cause of Fahreaus-Lindquist effect. Saffman [20] also showed that the effect of rotation of the sphere is to produce a lift force that acts in the same direction as the lift due to shear and that this contribution due to the rotation is a higher-order effect [21].

### Biological importance

Blood is an important biological example. Consisting mainly (45% volume) of relatively massive red blood cells (erythrocytes), it also contains small spheroidal cellular fragments called platelets (or thrombocytes). Their aim is to move with the flow in the vicinity of a blood vessel wall and to stick to possible injury to prevent bleeding. Blood platelets adhere to injured tissues to initiate the primary haemostasis. For a platelet to attach to the exposed subendothelium of a blood vessel, the adhesion force must overcome the drag force from the blood flow. The adhesion of platelets is to a great extent governed by their motion regimes in a shear flow and hydrodynamic interaction with erythrocytes and wall. The non-spherical platelet shape brings an additional complexity to the adhesion mechanics. The flipping motion of a blood platelet under the action of a simple shear flow over a flat substrate is discussed in [22]. Mody and King [23, 24, 32] presented a detailed study of the flipping motion of tethered [24] and free platelets [23, 32] near a plane wall, and developed a pertinent hydrodynamic model. In their work the influence of a flat wall on platelet aggregation was investigated. It was found that platelet collision frequency and aggregation mechanics depend strongly on particle shape and a distance from the bounding wall.

### Micropatterned surfaces and microfluidics

Sorting of micron-sized solid particles, blood cells and polymer molecules, isolation of biological cells or viruses from complex mixtures are important issues for a variety of material science and biomedical applications. For example, chaotic mixing and fractionation of suspensions can be effectively performed in microfluidic channels with special anisotropic pattern. Manipulation with biological cells in suspensions is a central problem in a variety of biomedical applications, including microfluidic devices dealing with whole blood samples. The surfaces of microfluidic channels should provide hemo-compatibility together with efficient transport characteristics. Usual design considers smooth plane wall. Roughness-induced transport properties, such as super-hydrophobicity and transverse flow generation, are being intensively implemented in microfluidics nowadays [25–27]. For instance, recently micro-scale roughness and grooved surfaces have been proposed to separate inertial particles [28].

Several approaches were made to describe an impact of surface roughness to hydrodynamic interactions of microparticles and adhesion. These works mainly focused on nano-scale roughness, which typical dimensions are much smaller that that of the microparticle [29]. The sorting of particles in microfluidics, on contrary, was attributed to microscale grooves with period much bigger than the diameter of the particles or considered only spherical particles [28]. What happens if the roughness is of the same dimension as the microparticle itself, is the issue addressed in the present paper.

We devote our analysis and simulations to the case of rigid spheroidal inertial particles of typical size of 4–8 microns flowing in the vicinity of a parallel-grooved surface with cylindrical grooves of radius of 4 *μ*m.

## Materials and methods

### Computer model

The computer model was based on a combination of the Lattice Boltzmann method [30] to calculate hydrodynamic flows with Lagrangian Particle Dynamics implemented in ESPResSo open-source software [31]. Lattice Boltzmann scheme D3Q19 was used. The following parameters were used to set up the fluid component in the model: the lattice period Δ*x* = 1 *μ*m, time step Δ*t* = 1 *μ*s, kinematic viscosity and density of the fluid *ν* = 1.0 *μ*m^2^/*μ*s, *ρ* = 1.0 kg/m^3^. A single relaxation time was used for the collision step. The value of the relaxation time was calculated from the desired value of kinematic viscosity $\tau =0.5+\nu /(c_{s}^{2}\Delta t)$, where $c_{s}^{2}=\left(1/3\right){\left(\Delta x/\Delta t\right)}^{2}$ is the lattice speed of sound. The simulation box size was 120x32x20 *μ*m^3^.

**Fig 1:**
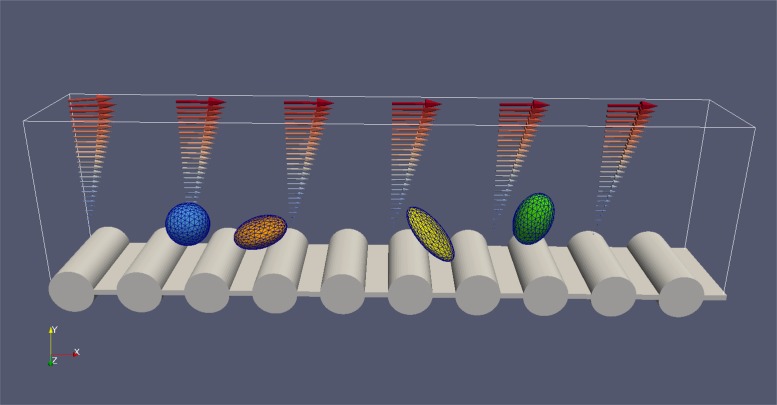
Overview of the model setup.

**Fig 2:**
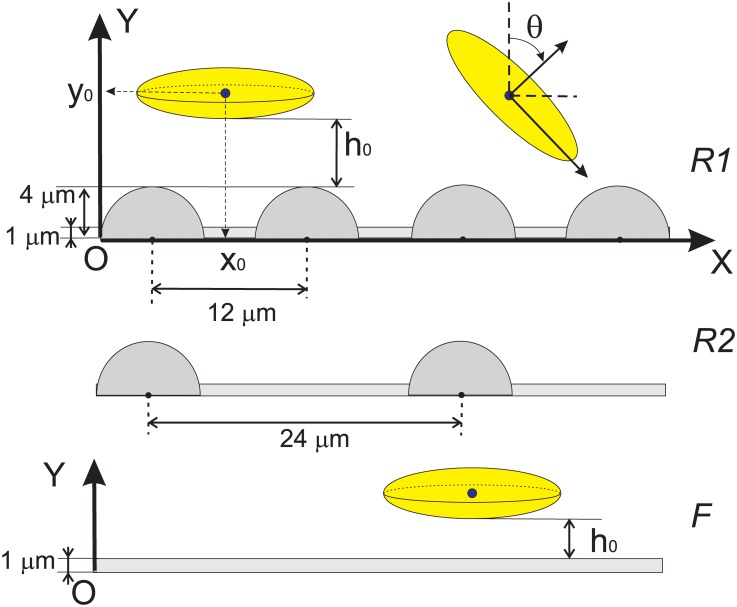
Scheme of the modelled system: Rough surfaces 1 and 2 (denoted R1 and R2 respectively) are comprised from an array of cylinders of a 4 *μ*m radius. The axes of cylinders are oriented parallel to *Z* axis and located at coordinate *Y* = 0 *μ*m. The array of cylinders is periodic in *X*-direction with a period 12 *μ*m for R1 and 24 *μ*m for R2. Flat surface (denoted F) corresponds to the case when radius of cylinders is set to zero. Here *h*_0_ is the initial distance between particle and the surface, and *x*_0_ is the initial position of particle’s centroid along the direction of the fluid flow. The angle *θ* is measured between *Y* axis and particle’s main symmetry axis.

The micro-relief was introduced as a periodic array of cylinders (Fig 1). The radius of each cylindrical obstacle was 4 *μ*m, the period of the texture was 12 *μ*m and 24 *μ*m for textures 1 and 2 respectively. Before each simulation run, the microparticle was placed near the wall at a certain distance *h*_0_ from the top of the roughness or from the flat surface. We impose the no-slip hydrodynamic boundary condition on the wall. The initial gap between particle and surface *h*_0_ was measured from this no-slip hydrodynamic boundaries as shown in Fig 2. The constant shear rate (Couette flow) was established due to imposed velocity boundary condition on the top plane of the simulation box, Fig 1. Three types of surfaces were studied in the present work in Fig 2: flat, texture 1 (R1) and texture 2 (R2).

For setting up the hydrodynamic boundaries in this work was used the “link bounce back” method, which is described in the literature [30]. The boundary shape is presented as a raw unstructured triangulated surface by the unit normal and vertices of the triangles in a three-dimensional Cartesian coordinate system. The lattice nodes are determined once during initialisation whether they are boundary or fluid nodes. If one creates a cylinder with a certain radius around a certain point all nodes inside this cylinder are flagged “wall”, while the outermost are flagged “fluid”. The hydrodynamic no-slip boundary appears halfway between two neighbouring nodes. Therefore, the no-slip hydrodynamic boundary for the flat surface was located at *Y* = 1 *μ*m due to appropriate choice of locations of Lattice nodes: 0.5 *μ*m, 1.5 *μ*m, 2.5 *μ*m, etc. The same holds for cylindrical grooves (see S1 Fig): so that the velocity of the fluid vanishes at the surface of cylinders. In the deepenings between cylinders the velocity naturally vanishes so this approximation was considered to be enough for the presented study. Notice curved streamlines between two cylinders (S1 Fig). However, in this work initial placement of the particles in contact (*h*_0_ ≪ Δ*x*) with surface was avoided. Following [32], a very short-range repulsive force between the surfaces of wall and the particle was included in the model to account for short-range interactions that boundaries from overlapping. The soft sphere potential (with origin at the no-slip hydrodynamic boundary) was introduced *U*_*s*_ = 0.001 ⋅ *r*^−1.2^. The cut-off distance was 0.2 *μ*m. Similar short-range repulsion force has been used in a number of prior work for modelling interactions and collisions of biological cells [23, 32]. The on-site velocity boundary condition on the top plate of the simulation box was imposed according to [33] (see Eq (6.40)-(6.43) therein).

Each spheroidal microparticle was represented by a mesh of 304 Lagrangian surface points (or LSPs) interconnected via elastic bonds. Molecular dynamics (MD) approach was used to calculate trajectories of LSPs. The LB scheme and the particle coordinate update scheme were not synchronized: in each LB time step, 10 MD steps were performed. This allowed to the speed up the simulations. In this implementation, the coupling between the fluid and the particles was provided via the viscous-like force acting between LB lattice nodes and membrane surface point [30, 31]. This force exerted on membrane point, analogous to the Stokes formula, was taken to be proportional to the difference of the velocity *v* of the LSPs and the fluid velocity *u* derived from the LB lattice at the same position, *F*_*j*_ = *ξ*(*v*−*u*). And the opposite force −*F*_*j*_ was transferred back to the fluid. The rigidity of particles was maintained by high values of elastic constants in the particle constitutive model: stretching elasticity constant *k*_*s*_ = 10.0 nN/*μ*m, bending elasticity *k*_*b*_ = 10.0 nN ⋅ *μ*m, global and local area conservation constants *k*_*ag*_ = *k*_*al*_ = 10.0 nN/*μ*m, and volume conservation constant *k*_*v*_ = 10.0 nN ⋅ /*μ*m^2^. With such a choice of parameters in all presented simulation runs the relative membrane deformation (controlled by observing the lengths of particle’s main axes) did not exceed 1% in the range of shear range from 50 /s to 300 /s.

Five different shapes of rigid microparticles were considered in the presented study. The aspect ratio was varied so that the volume of the particle was maintained. Radius of a spherical particle was 4 um, the sizes of other particles were adjusted so that they have the same volume as sphere. So, for *a*/*c* = 1: 2 spheroid the longer semiaxis *c* = 5.04 *μ*m and the lesser one *a* = 0.5 ⋅ *c* = 2.52 *μ*m, etc. The parameter of friction *ξ* was adjusted from the calibration procedure described in [31], as well as the masses of LSPs. Mass of each LSP point of a particle surface was calibrated with a “viscous deceleration” benchmark, proposed in [31]. Within this approach we set the initial velocity of the particle in unbounded layer of fluid and track its dynamic. Due to coupling between fluid and LSPs coupling, fluid exerts a drag force and slows down the particle. The number of LSPs per each mircoparticle was set to a constant value (304 points) with compromise between computational efficiency and accuracy. The mass of each LSP *m*_*ib*_ and the friction coefficient *ξ* have been changed in order to reproduce analytical solution *V*(*t*) = *V*_0_ ⋅ exp(−6*πμaKt*/*M*) in the simulation (see S2 Fig). Here *μ* = *ν* ⋅ *ρ* is the dynamic viscosity, *a* is the particle size (bigger semi-axis), *M* = 304 ⋅ *m*_*ib*_ is the particle mass and *K* is the form-factor. The following values were found to give a satisfactory convergence: *m*_*ib*_ = 10.0 fg, *ξ* = 0.7 nN ⋅ *μ*s/*μ*m.

### Validation

**Fig 3:**
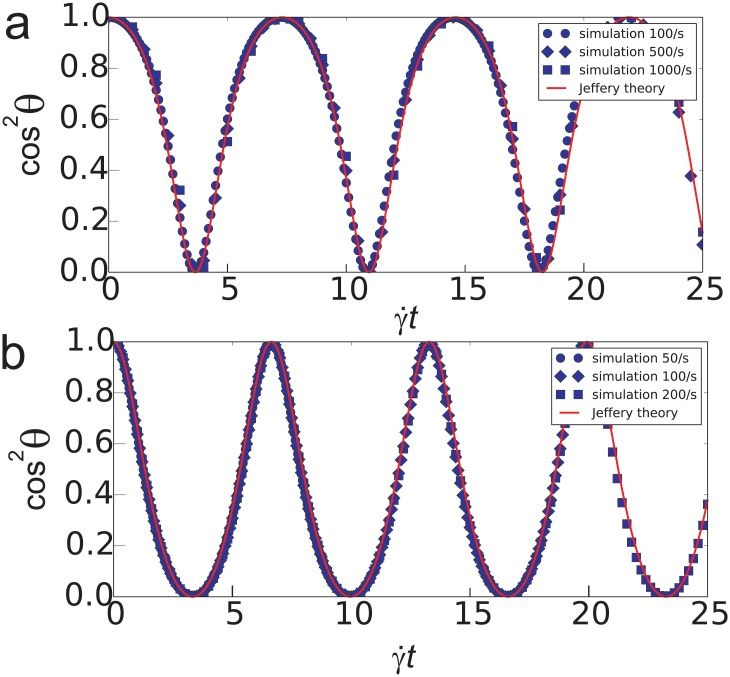
Validation test to reproduce Jeffery’s orbits [8] for spheroidal particles in non-bounded shear flow of a viscous fluid for oblate *a*/*c* = 1: 2 (a) and prolate 3: 2 (b) particles. Lines correspond to analytical solution and dots—to simulation results.

In order to validate the model, there were used two principal testcases: (i) Jeffery’s orbiting of a freely suspended spheroid in an unbounded shear flow [8] and (ii) the motion of a sphere in a shear flow near a flat wall [34]. The following analytical solution was obtained by Jeffery [8] for the rotation of the main symmetry axis:

$$\begin{array}{c} \tan\theta =\frac{1}{\Gamma }\tan(\frac{\Gamma t\cdot \dot{\gamma }}{1+\Gamma^{2}}), \end{array}$$ (1)

where Γ = *c*/*a* with *a* being the particle semi-axis in the direction of the highest symmetry. Each particle was initially oriented such that their axis of symmetry coincided with the Y axis of the coordinate system. Fig 3 shows the plots of the square cosine of the angle *θ* between axis and Y direction of the coordinate system: cos^2^ *θ* = (1 + tan^2^ *θ*)^−1^. The satisfactory agreement between simulation and theory was observed for oblate and prolate spheroids in shear rates ranging from 50 to 1000 /s.

Another validation test relies on the work of Goldman et al. [34], where numerical data for sphere free-rolling motion in a shear flow near a plane wall were presented along with the following asymptotic analytical expressions:

$$\begin{array}{c} \frac{V_{x}}{\left(h_{0}+a\right)\dot{\gamma }}\approx 1-\frac{5}{16}{\left(\frac{a}{h_{0}+a}\right)}^{3},\;h_{0}>a, \end{array}$$ (2)

$$\begin{array}{c} \frac{V_{x}}{\left(h_{0}+a\right)\dot{\gamma }}\approx \frac{0.7431}{0.6376-0.200\cdot \ln(h_{0}/a)},\;h_{0}\ll a. \end{array}$$ (3)

**Fig 4:**
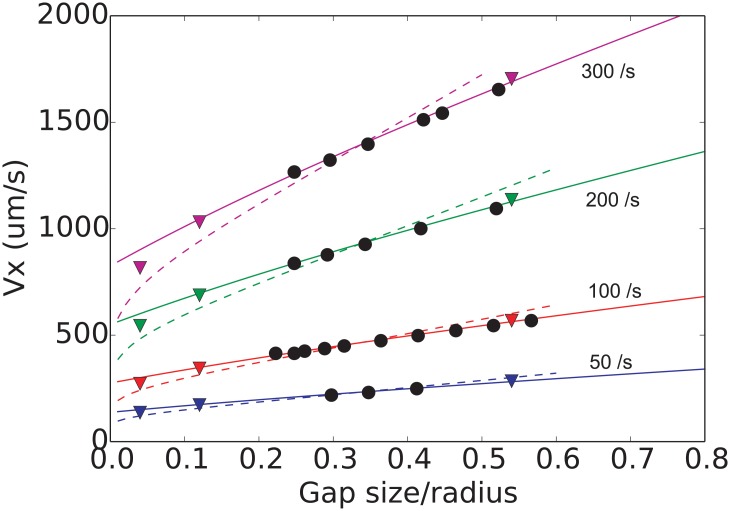
Sphere near wall case used for the validation of the computer model. The velocity of sphere is plotted vs. initial position of the sphere for 4 shear rates. The dashed line and solid line correspond to asymptotic analytical solutions by Goldman et al. [34], and triangles—to numerical results obtained therein. Black circles represent the results of our computer simulations.

The sphere was placed with different gap sizes *h*_0_ with respect to hydrodynamic no-slip boundary. The results are presented in Fig 4. For this case the repulsive interaction between the sphere and the surface were switched off to observe pure hydrodynamic effects. Again the results are in good agreement with theory.

## Results

### Non-inertial motion (100/s)

Here we focus on the case of relatively small shear rate 100 /s. That corresponds to the absence of any inertial effects on the motion of particles during our 500 *μ*s-simulations. For every set-up we provide the “control” simulation with a flat wall. In the beginning of each simulation run the particles were placed to a certain distance *h*_0_ from the hydrodynamic boundary of the bottom surface. The gap *h*_0_ between surfaces of the bottom wall and the particle was the same for flat and rough surfaces. The value of *h*_0_ was varied during the study to analyze the dependence of initial placement on motion of particles. The initial X-coordinate (unless explicitly specified) was set to *x*_0_ = 5 *μm*.

**Fig 5:**
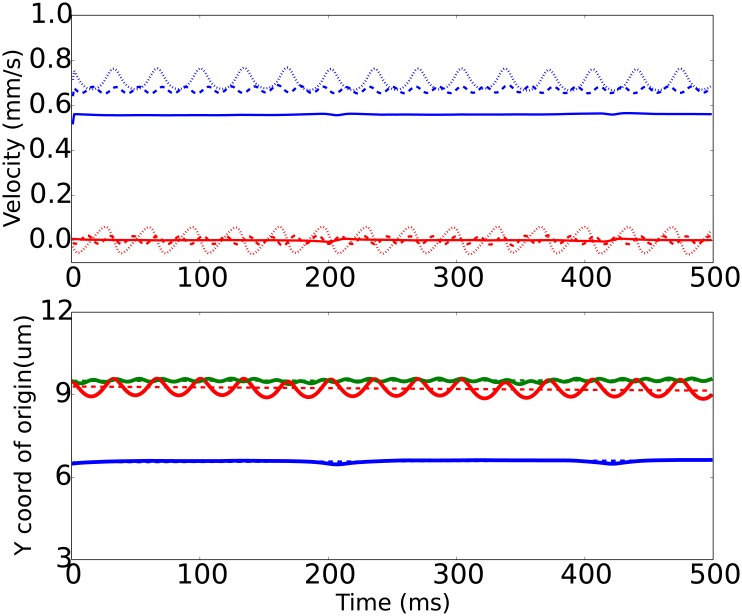
Time courses for a microparticle’s velocity components (top panel) and vertical coordinate of its center of mass (bottom panel) obtained from computer simulation for a sphere in a shear flow of 100 /s. Top panel: blue lines correspond to translational velocity of a microparticle *V*_*x*_, red—vertical (lift) velocity *V*_*y*_. Solid, dashed and dotted lines—respectively correspond to flat, rough 1 and rough 2 surfaces. Bottom panel: blue, green and red solid lines correspond to flat, rough 1 and rough 2 surfaces; dashed lines are linear fits to the simulation results of the particle’s center during the timespan from 200 to 500 ms. Initial position of particle in this case *h*_0_ = 1.5 *μm*, *x*_0_ = 5 *μm*.

**Fig 6:**
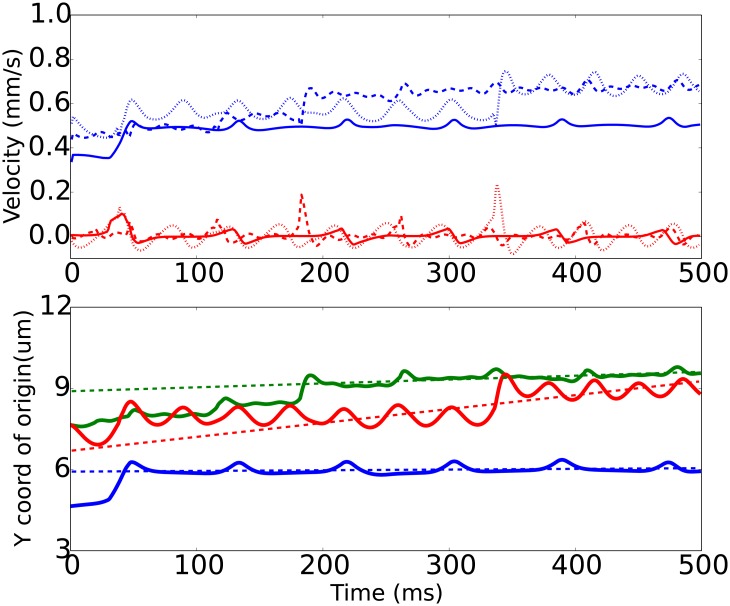
Same as Fig 5, but for oblate 1:2 spheroids (shear rate $\dot{\gamma }=100/s$, *h*_0_ = 1.0 *μm*, *x*_0_ = 5 *μm*).

**Fig 7:**
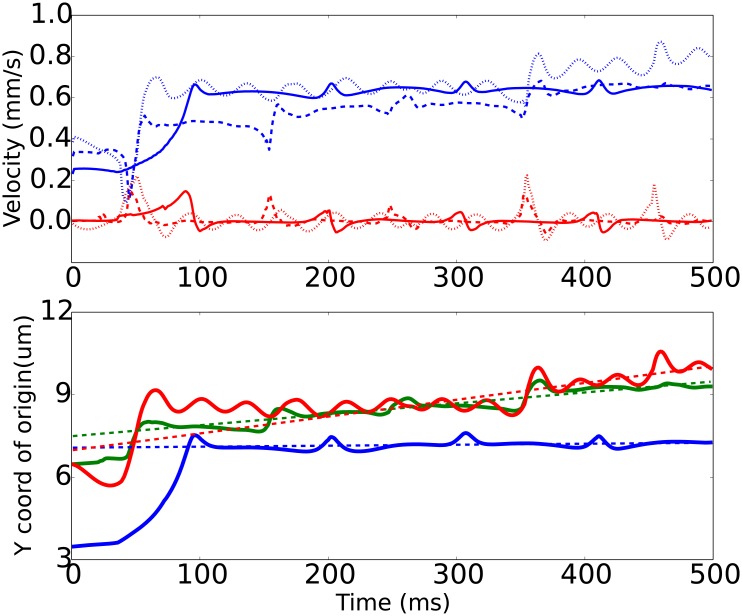
Same as Fig 5, but for oblate 1:4 spheroids ($\dot{\gamma }=100/s$, *h*_0_ = 1.0 *μm*, *x*_0_ = 5 *μm*).

**Fig 8:**
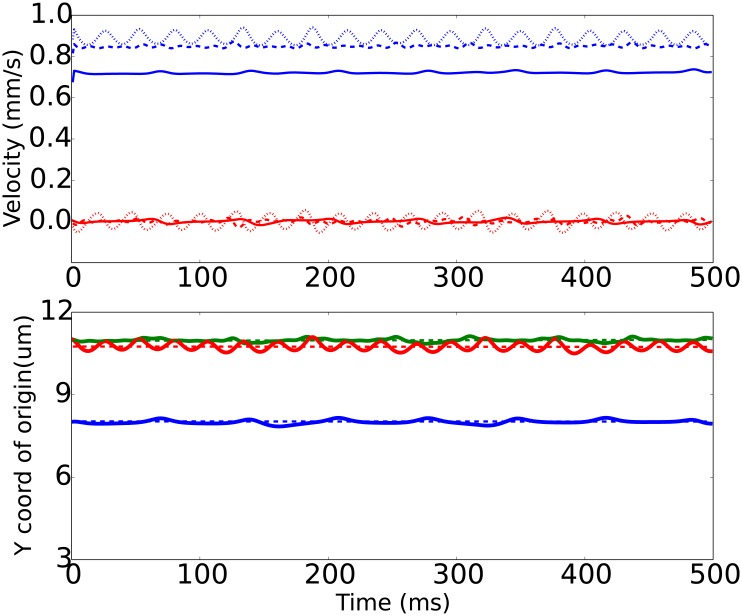
Same as Fig 5, but for prolate 3:2 spheroids ($\dot{\gamma }=100/s$, *h*_0_ = 1.8 *μm*, *x*_0_ = 5 *μm*).

Typical time courses for the particle centroid position and velocity are shown in Figs 5–8. Here the vertical Y-coordinate was measured from the base surface *y* = 0. We see that the motion pattern depends on the shape of particle alters as well as on the surface relief. General observation is that after a certain time all particles reach the steady distance from the surface and the quasi-steady (average) translational velocity *V*_*x*_. This distance (as well as *V*_*x*_) depends on particle shape and relief of the surface.

The spherical particle (Fig 5) tends to maintain the distance to the wall, and its motion is mostly steady. Is case of rough surfaces, the origin oscillates near a mean position equal to the initial distance to the base of the rough plane. In case of the rough surface 2 (denoted R2) the oscillations are two times less frequent but their amplitude is much bigger. The comparison with visualization confirms that these oscillations are caused by the roughness, which is less frequent in case of R2 (compared to R1). The apparent increase of the amplitude of the origins’ oscillations in Y-direction is explained by the fact that the particle falls into hollows between two obstacles deeper in case of a less frequent texture R2. Thus, dense texture of R1 surface effectively pushes the sphere further from the base plane into the bulk fluid. Interestingly, the X-velocity (along the flow) is the greatest for the sphere in case of the R2 surface, although the position of the particle’s origin is higher (further) from the surface. This is due to the fact, that the more frequent roughness R1 slows the fluid flow in the vicinity of the wall as a result of an increased fluid-wall contact area and consequent increase of energy dissipation. Thus, the R2 surface is somewhat optimal for the case of spherical particles—it provides reasonable particle push-out from the wall, yet does not slow the fluid too mush.

The oblate spheroidal particles, or platelets, show more interesting behaviour, Figs 6 and 7. As a consequence of their shape they naturally perform flipping (tumbling) motion in shear flow [8]. The time courses for oblate spheroids near flat wall are qualitatively similar to results of Mody et al. [32] When this motion regime interferes with periodic surface relief, the platelet moves in a tumbling with a series of consequent “jumps” from the rough surface. These jumps are caused by the mechanical interaction of platelets with the periodic array of cylindrical obstacles.

As for the velocity *V*_*x*_ of the translation along the flow, again the rough wall with longer periodicity was the fastest for oblate spheroids (for 1:4 spheroid *V*_*x*_ = 0.8 mm/s for R2 and 0.6 mm/s for R1), while for 1:2 spheroid both rough surfaces resulted in almost the same velocity equal to 0.7 mm/s.

The prolate spheroids (Fig 8) also move so that they resemble Jeffery’s orbits, however, their motion was somewhat similar to the sphere, since the steric interaction with roughness was minimized due to their initial placing procedure. But the flow with higher translational velocity *V*_*x*_ since their origin protrudes to the regions of faster fluid flows, resulting in greater drag forces and faster motion. Again, the R2-wall appeared to be the “fastest”, yet the R1-wall was the most repulsive during the simulation run.

### Initial jump

First thing that should be noticed is the initial “jump” from the wall exhibited by the oblate particles. The reason is understood: the rotational motion requires some space, so that the rim of the particle may pass above the surface. This steric effect lifts the particle origin to the distance approximately equal to the longer semi-axis from the local wall position. Here “local” means that as the micro-relief is present on the surface, the jump time end height may be dependent on when and where the particle bumps into the solid surface of the wall. As the particle moves along the relief of the rough surface, it may (i) slide over the obstacle while being in a horizontal orientation leading to minor lift, or (ii) hit the obstacle with its rim causing the maximal possible lifting. In the case (i) the rim falls into the the deepening of the relief, while case (ii) rises the origin into the regions of greater fluid velocities, dragging the top of the spheroid along the flow. For spherical particles this initial jump was not observed (when placed above the tops of the roughness) due to the shape: the sphere can freely rotate at any distance from the solid surface. For prolate spheroids there was no jump also because of the initial placement of particles, so that the longer symmetry axis was vertical. Therefore, the distance from the origin to the solid surface was always big enough, so that the rotation was possible.

**Fig 9:**
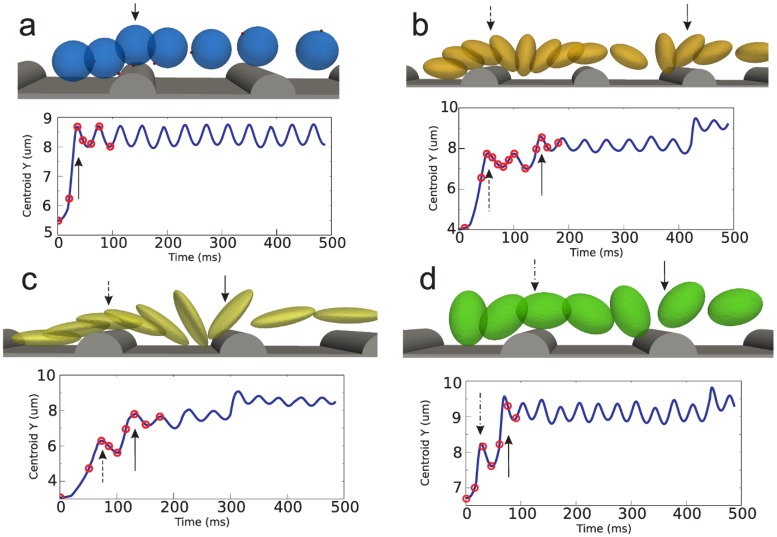
Illustration of different mechanisms of uptake of particles from the deepening between two grooves. Each panel (a-d) consists of a series of simulation snapshots and the plot of *Y*-coordinate of particle centroid vs. time. The red circles denote the respective moments of time, at which the snapshots were taken. The solid arrows mark moments of lift due to hitting the cylinder during flip-motion (panels b, c, d) and over-rolling (for panel a). The dashed arrows correspond to lift due to over-crawling (panels b, c). The dash-dotted arrow in panel d corresponds to over-hopping the obstacle.

In order to illustrate the mechanisms of initial repulsion of particles from micro-rough surfaces, we performed another series of simulation runs at shear rate 100 /s. For that case the particles were placed in the deepening between two cylindrical grooves of surface R2, so that the minimal distance between the particle’s surface and the lower hydrodynamic boundary was 1 *μm* for all particles. The results summarized in Fig 9 demonstrate different mechanisms of initial repulsion (or “jump”) due to mechanical interactions between particles and cylindrical grooves. It could be noticed that oblate spheroids perform “jump” in two steps. First, they tend to over-crawl the obstacle (marked with dashed arrows in Fig 9b and 9c). After this flipping motion sets in, and the consequent repulsion is associated with hitting the tops of the grooves during rotation. Prolate spheroid hops over the first obstacle, starts rotating and then hits the cylinder (Fig 9d). For spherical particles over-rotation was the only option due to their shape (marked with solid arrow in Fig 9a). Notice that this initial jump is caused only by mechanical collisions between microparticles and rough surfaces.

### Steady non-inertial motion

**Fig 10:**
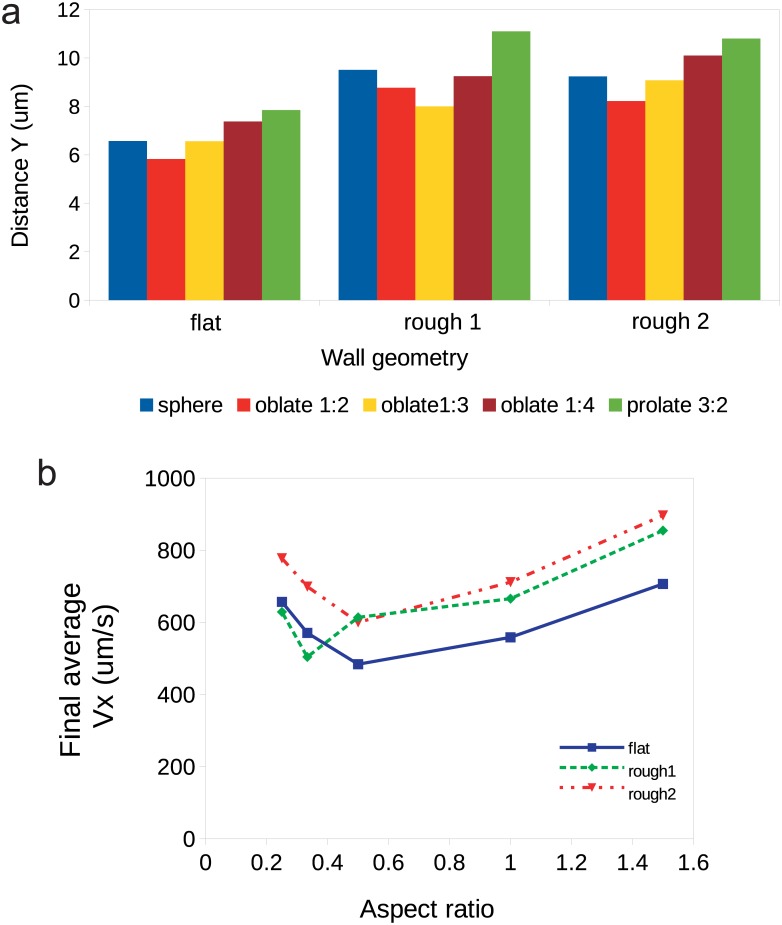
(a) Maximal (equilibrium) vertical distance *Y* reached by the particles accounted from the base plane *y* = 0 after the initial jump in non-inertial regime (shear rate 100 /s). (b) Steady translational velocity of particles after the initial jump: solid line corresponds to flat wall, dashed and dash-dotted—to micro-rough surfaces 1 and 2. For all simulation runs here we set *h*_0_ = 0.5 *μm* and *x*_0_ = 5.0 *μm*.

The distance to which particles lift from their starting position was also dependent on the surface texture, as well as particle shape. We compare the effect of R1 and R2 walls on the maximal *Y* coordinate of the center of mass of particles reached during simulation in Fig 10. Here for all simulations we set *h*_0_ = 0.5 *μm* and *x*_0_ = 5.0 *μm*. Fig 10a shows the distance from the wall reached by each particle after the 500 *μ*s, which we consider as the quiescent distance to the wall. The velocity that corresponds to the final steady particle-to-wall distance is shown in Fig 10b. Generally, roughness results in a further repulsion of particles compared to the flat surface. One may suggest that roughness shifts the effective hydrodynamic boundary into the bulk fluid. We see that oblate spheroids have a tendency for a slower motion near all the studied surfaces, as their centroids are repelled to smaller distances from the effective hydrodynamic boundary.

### Influence of the initial positioning

**Fig 11:**
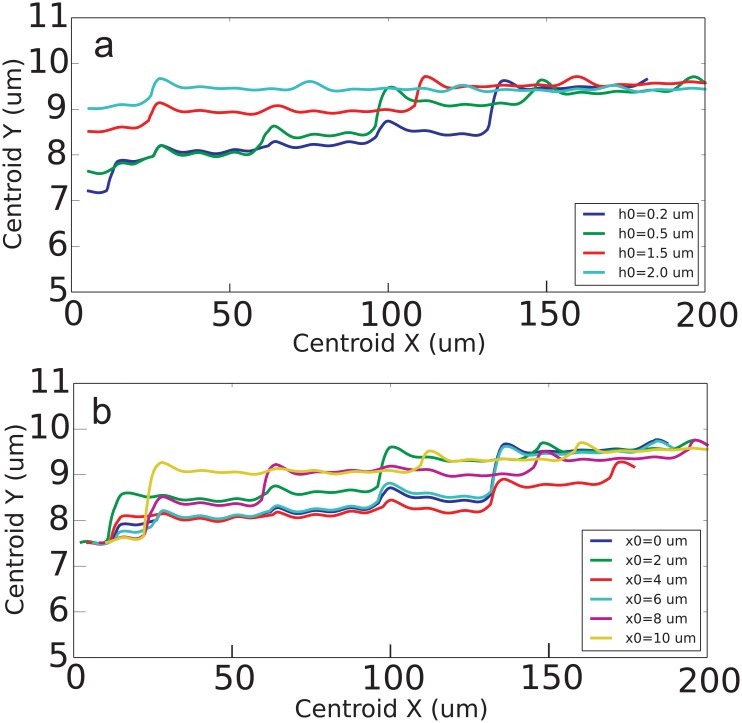
(a) The trajectories of 1:2 spheroids moving near R1 rough wall in 100/s shear flow starting from different distances to the wall *h*_0_ and the same *x*_0_ = 5 *μm*. (b) The trajectories of 1:2 spheroids moving near R1 rough wall in 100/s shear flow starting the same distance to the wall *h*_0_ = 0.5 *μm* and different *x*_0_.

In order to study how the initial placement influences the dynamics of particles and their steady position with respect to wall, another set of simulation runs was performed. The initial distance *h*_0_ and initial x-coordinate of particle’s center *x*_0_ were altered. The results for 1:2 spheroid moving over R1 surface in 100/s shear rate are presented in Fig 11. Generally, we see that particle reaches the same steady position regardless of its initial coordinate. The divergence was less than 5% for all studied *h*_0_ and *x*_0_. However, the time required to reach the steady distance and thus the uptake velocity depend on initial placement. The same in general holds for all studied shapes of particles.

## Discussion

In present study the effect of the roughness on the mobility of solid spheroidal particles in viscous fluid flow has been quantified depending on the aspect ratio of the spheroid and the geometry of a wall.

### Roughness-induced mechanism of lift

The mechanism of non-inertial roughness-induced lift from the wall can be understood from the following consideration (S3 Fig). When particles move near the rough surfaces they must overflow the obstacles. The rotation (or tumbling), being typical for sheared spheroidal particles, now in the presence of obstacles interferes with the series of bumps and falls due to the wall relief. This kind of motion represents the combination of two simultaneous periodic processes with the comparable frequencies (equal approximately to the frequency of Jeffery’s orbiting). When the particle’s origin is in the highest possible position, then the particle can hop over the obstacle, being dragged by the streaming fluid. This motion pattern is more important for an oblate particle: it can dive into the deepening of the relief with their rim, exposing the rest of its surface to the flow, just as a sail. The flow flips the particle forcing it to “land” on the wall with its flat and wide “belly”-surface. This causes the deceleration of the rotative motion due to the lubrication forces between particle and wall. The roughness compels the particle to experience this hydrodynamic repulsion at a greater distance from the wall, thus lifting the instant axis of particle’s rotation each time the particle hits the roughness. If due to a coincidence of the two periodic processes mentioned above the particle hits the roughness (cylinder) with its rim, then the maximal lift effect is observed, since the instant rotation axis jumps to the top of the surface texture. Thus non-inertial lift occurs as a series of jumps in cases of rough surfaces. There is no surprise that the period of the texture of the surface influences this process quantitatively.

### Particle shape

We see that spheres and prolate spheroids are less susceptible to the roughness, unlike oblate particles. For non-inertial regime of motion (100 /s) we observed that oblate 1:2 particles jumped to the smallest equilibrium distance from the wall in cases of flat wall and sparse relief (rough 2), while for the dense relief (rough 1) the smallest jump was demonstrated by the oblate 1:3 spheroids. The same holds for the translational velocities, as they increase with the distance from the wall.

Another important notion is the dependence of initial uptake (“jump”) mechanics on the particle shape. This effect may have important biological consequences. For instance, consider blood platelets, which are meant to adhere and aggregate at the injured wall of blood vessel. The over-crawling regime of motion, typical for oblate spheroids, may provide greater surface for ligand-receptor binding than the tumbling motion or rolling. Thus platelets have greater chances to adhere compared to spheres and prolate spheroids.

### Roughness periodicity

Overall, we see that R1 wall, that is characterized by a smaller period, is generally more repulsive than the R2 wall and flat wall. However, the roughness does not only affect the particle’s motion pattern, but also alters the hydrodynamic flows in the near-wall region. The smaller the period of roughness, the bigger the interface area between fluid and solid wall, the greater the energy dissipation due to viscosity. Consequently, the sparse roughness of R2 wall causes less deceleration of particles than R1 surface.

Our calculations show that, depending on the shape, the particles may be slowed down by the deepenings of the relief. This trapping is especially important for oblate spheroidal particles with combination of a sparse relief (wall R2). In the latter case the particle may fall into the deepening of the surface texture, where the local fluid flows are hindered by the roughness, and this would lead to the consequent loss of particle’s momentum and decrease of hydrodynamic forces.

### Inertial effects

Most of the effects reported in the present paper are non-inertial. The inertial effects were not observed during 500 *μ*s simulations at shear rates <100/*s*, since the lift forces, induced by the fluid inertia [15, 21, 35], scale with *Re*_*G*_ number as [20]:

$$\begin{array}{c} F_{Lift}\approx 6.46a\mu R_{G}^{1/2}V_{slip}, \end{array}$$ (4)

and therefore require very long time to become evident, since *Re*_*G*_ = 16 ⋅ 10^−4^ ≪ 1 for our system. We see in this case that particles lift to the equilibrium position *Y*, in which they can perform the tumbling motion (combination of translation and rotation) in a shear flow without touching the surface. In this case fluid inertia was not involved into repulsion. Only the mechanical interactions with surfaces were involved. Based on the interplay between tumbling or rotational motion caused by the vorticity of the shear flow and the collisions with obstacles caused by the translational motion of the particle, this repulsion still may be considered as a hydrodynamic effect. For the particle inertia effects [6, 7], the Stokes number $St=t_{0}\dot{\gamma }$ defined as the ratio of the characteristic time of a particle to a characteristic time of the flow governs the manifestation. In our case characteristic viscous time *t*_0_ ≈ 100 *μ*s (S2 Fig), thus for 100/s shear rates *St* = 0.01 ≪ 1. For that reason, in our simulations we could not observe the drift of particles towards the wall as in Ref. [7]. However, the onset of transition to the log-rolling regime [6] was found at around 400–450 *μ*s for oblate spheroids observed by a small nutation of their symmetry axis. It would be instructive to address the effects of fluid and particle inertia in further systematic studies.

## Conclusion

Isolation of biological cells and/or microparticles from mixtures and suspensions is a central problem in a variety of biomedical applications. This problem is of an immense importance for microfluidic devices manipulating with whole blood samples. It is instructive to know how the mobility and dynamics of blood cells is altered by the presence of micrometer-size roughness on walls. The presented theoretical study addresses this issue via computer simulations.

We have shown that the periodic micro-scale roughness of a length scale comparable to the sizes of spheroidal microparticles can change quantitatively the periodic tumbling motion of the latter ones. This mechanical interaction repulses the particles from the surface and affects their mobility. The effect is strongly sensitive to the shape of particle and is much more pronounced for oblate spheroids (platelets). This study revealed distinctive patterns of motion for oblate and prolate particles during the initial jump phase. In this work we do not study the influence of particle deformability on its motion near rough surfaces. This phenomenon is known to have a significant impact on interactions between red blood cells and vessel walls and should be addressed in future works. Here we focus only on the dynamics of rigid objects, being inspired by blood platelets, which are practically non-deformable (at least in non-activated state) and move predominantly in the near wall region of the blood vessel. Hopefully, the presented findings would shed light on biomechanical details of motion of blood platelets (thrombocytes) near rough surfaces. Therefore we aspire for application of micro-rough surfaces as an anti-thrombotic coatings of implantable devices.

## Supporting information

S1 Fig To the illustration of hydrodynamic boundary conditions at rough surfaces. (PDF)

S2 Fig Particle parameters calibration. The initial velocity 0.1 *μ*m/*μ*s was set to all LSPs of a micro-particle. Due to viscous drag the particle slowed down. This figure shows typical results of simulation (dots) compared to theory (line) for a spherical 4-*μ*m particle. The LSP mass *m*_*ib*_ = 10.0 fg and viscous coupling coefficient *ξ* = 0.7 nN ⋅ *μ*s/*μ*m give adequate results.
(PDF)

S3 Fig Illustration to the roughness-induced hydrodynamic lift mechanism. Panels A and B demonstrate flipping motion of spheroidal particles; C and D show the corresponding time courses for velocities; panels E and F show the increase of particle’s *Y*-coordinate after the flip.
(PDF)
