# Supplementary figures and images for: Hydrodynamic repulsion of spheroidal microparticles from micro-rough surfaces
Source: PLoS One. 2017 Aug 14;12(8):e0183093. doi: 10.1371/journal.pone.0183093 (PMC5555679; doi:10.1371/journal.pone.0183093)

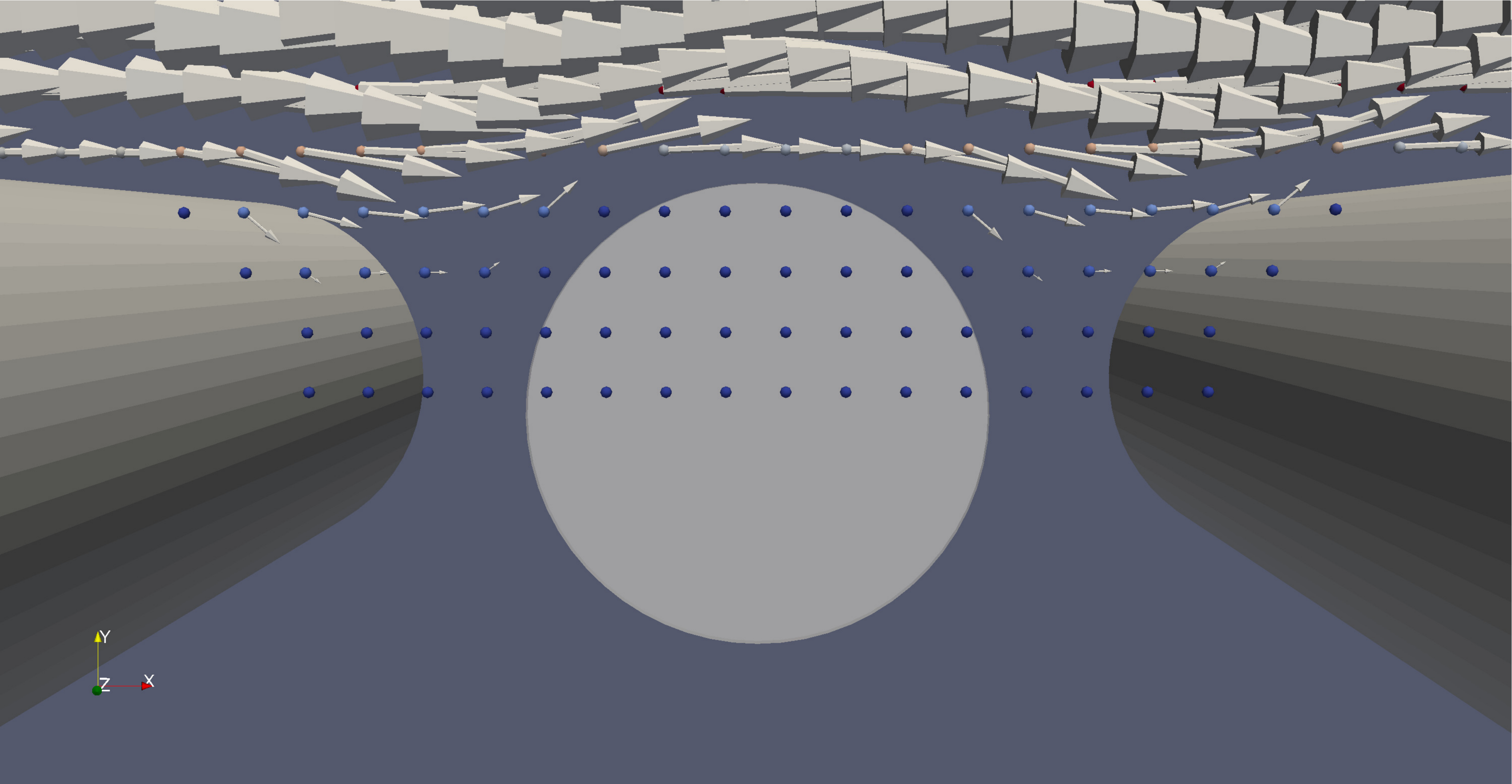

Supplement: S1 Fig — (PDF) [file pone.0183093.s001.pdf]

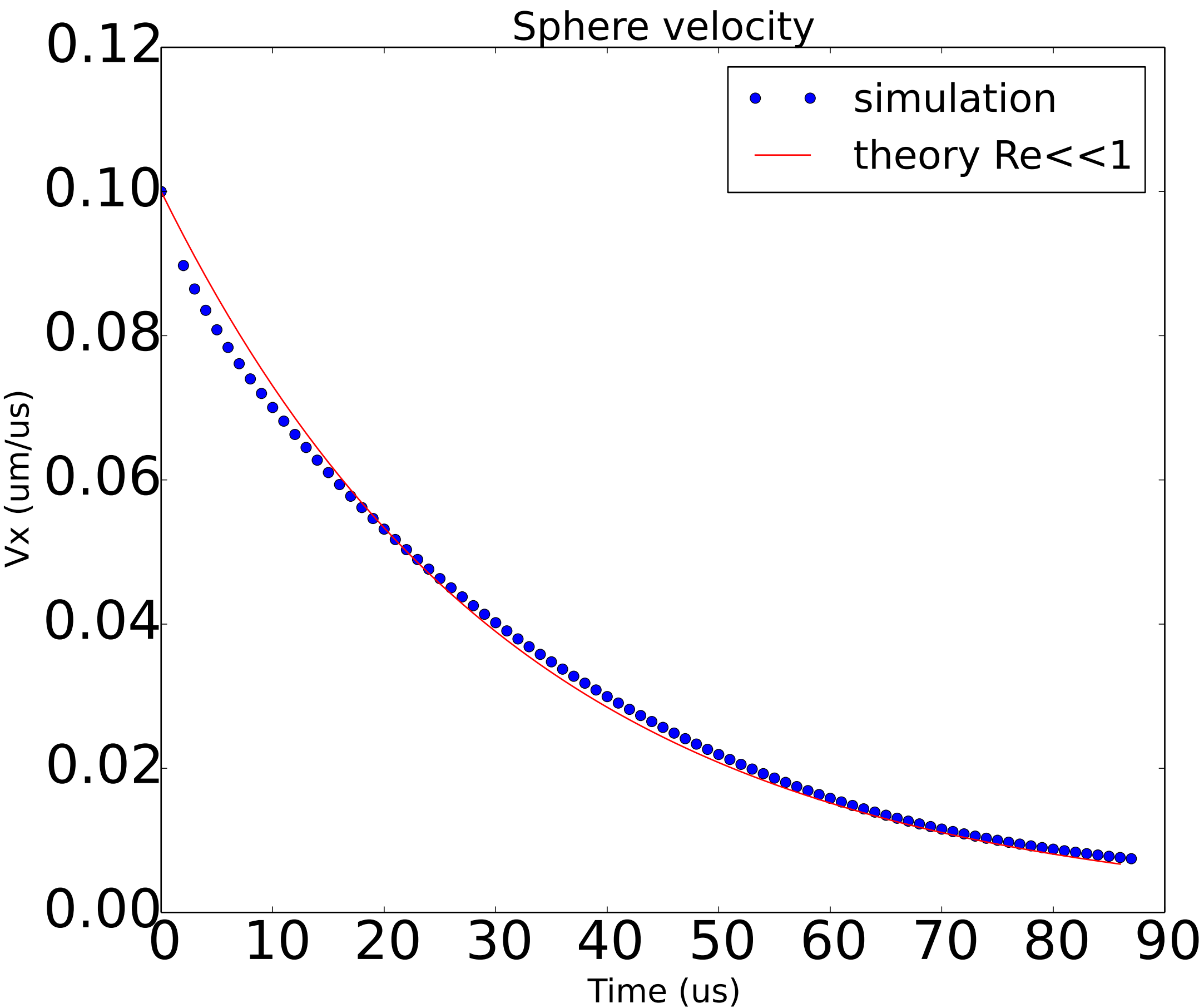

Supplement: S2 Fig — The initial velocity 0.1 μm/μs was set to all LSPs of a micro-particle. Due to viscous drag the particle slowed down. This figure shows typical results of simulation (dots) compared to theory (line) for a spherical 4-μm particle. The LSP mass mib = 10.0 fg and viscous coupling coefficient ξ = 0.7 nN ⋅ μs/μm give adequate results. (PDF) [file pone.0183093.s002.pdf]

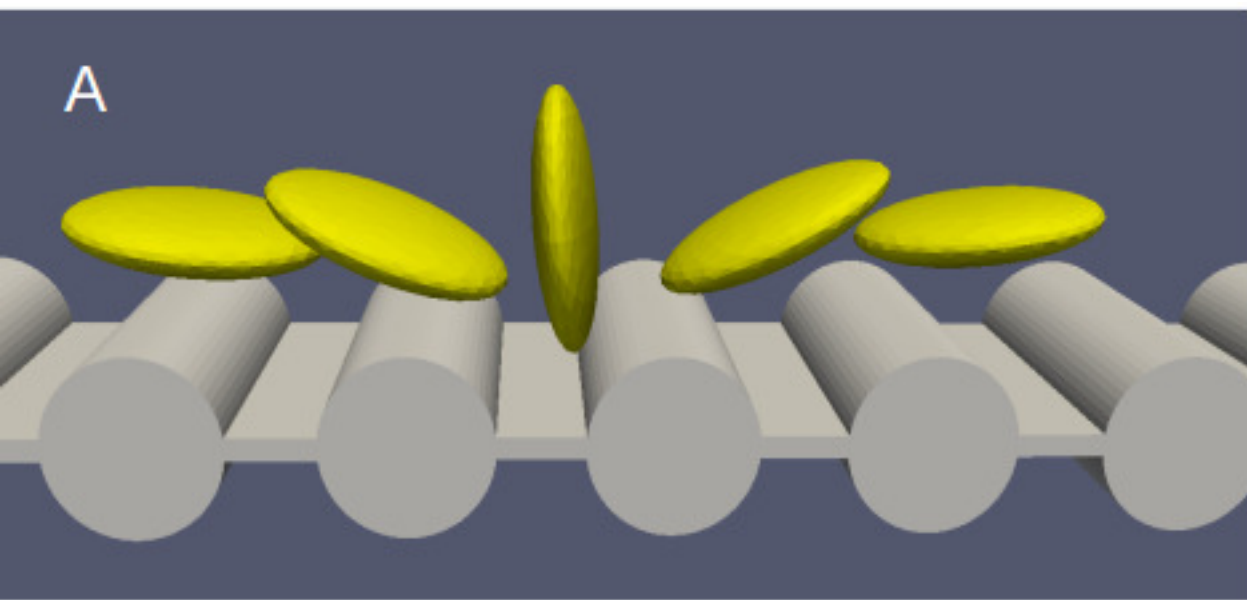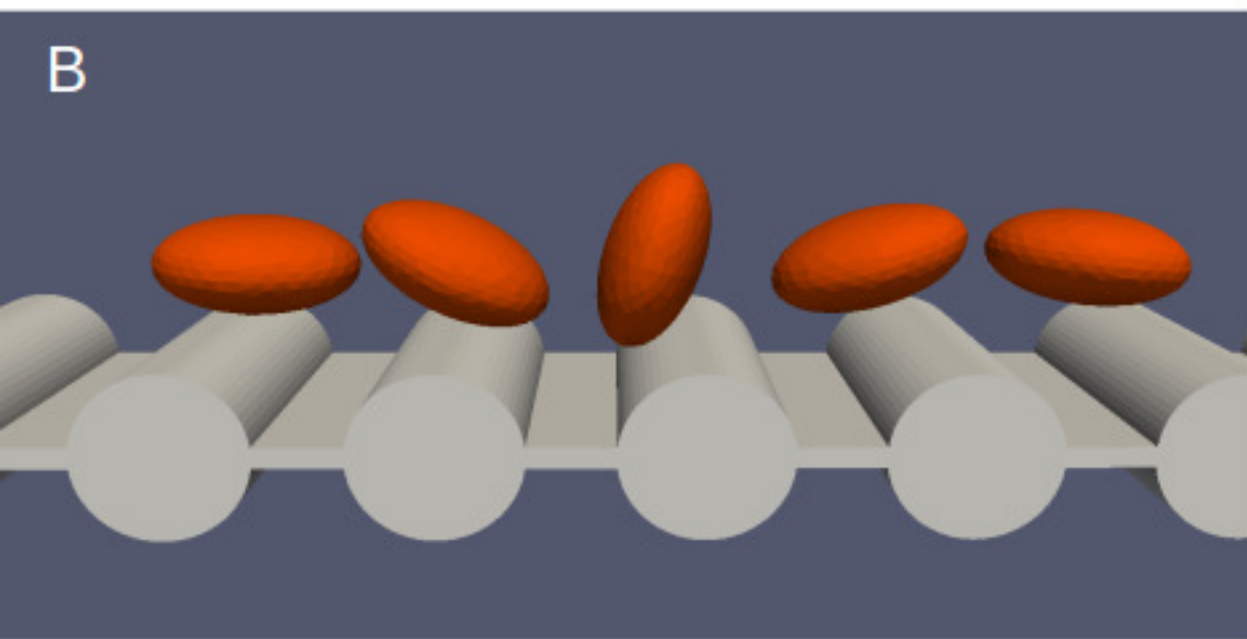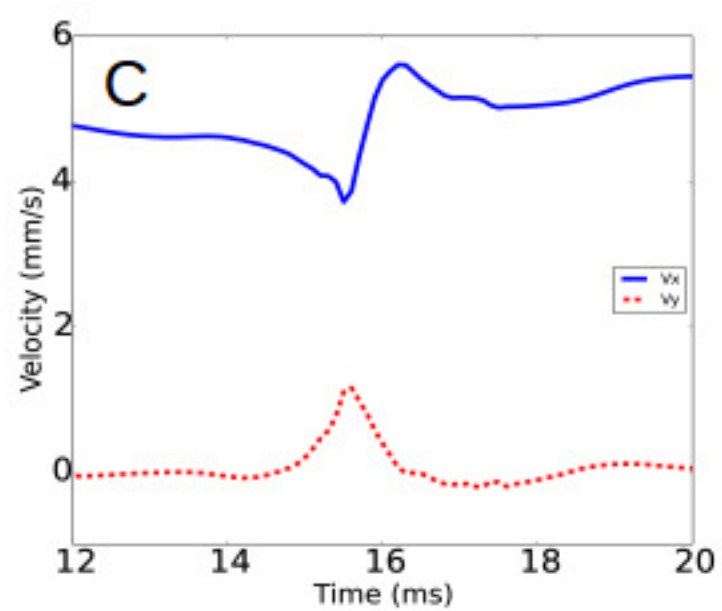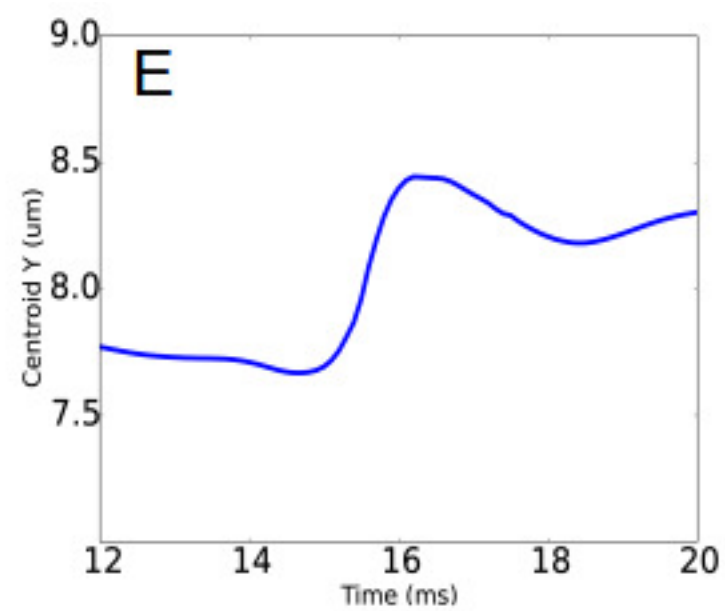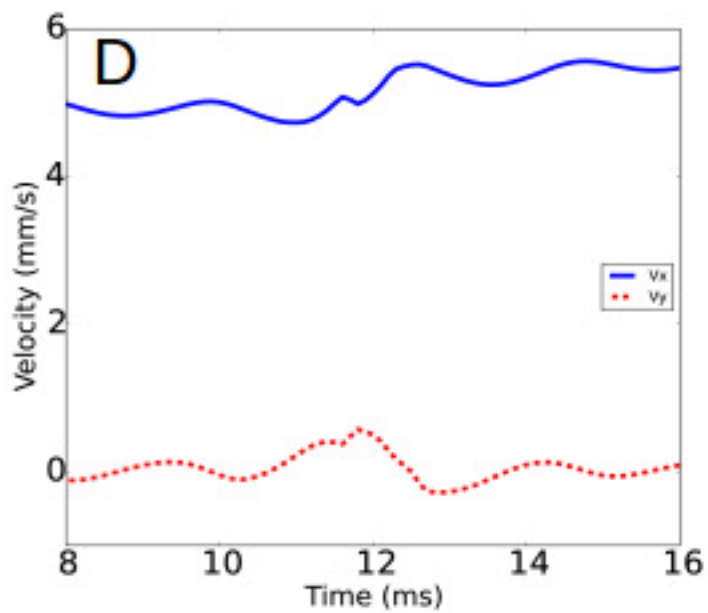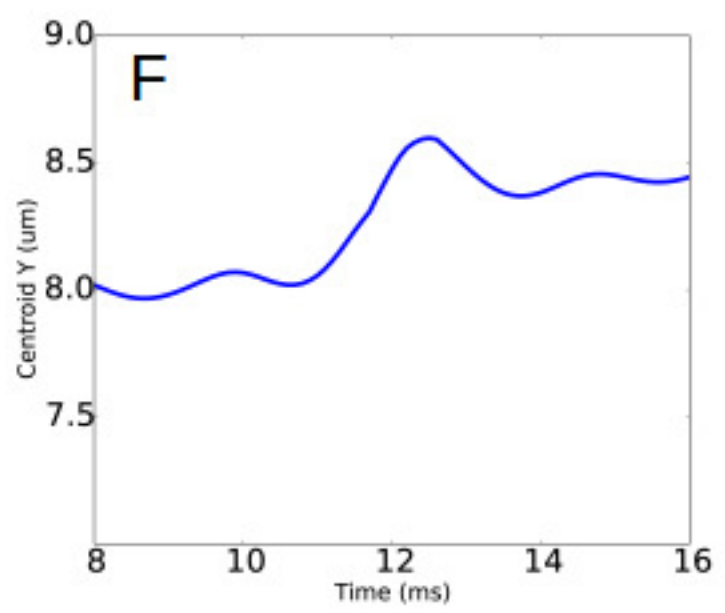

Supplement: S3 Fig — Panels A and B demonstrate flipping motion of spheroidal particles; C and D show the corresponding time courses for velocities; panels E and F show the increase of particle’s Y-coordinate after the flip. (PDF) [file pone.0183093.s003.pdf]
